# The Effect of Remote Digital Services on Health Care Inequalities Among People Under Long-Term Dermatology Follow-Up: Cross-Sectional Questionnaire Study

**DOI:** 10.2196/48981

**Published:** 2023-12-08

**Authors:** Serena Ramjee, Hanen Mohamedthani, Aditya Umeshkumar Patel, Rebeca Goiriz, Catherine A Harwood, Richard H Osborne, Christina Cheng, Zeeshaan-ul Hasan

**Affiliations:** 1 Barts and the London School of Medicine and Dentistry Queen Mary University London London United Kingdom; 2 Dermatology Department Barts Health NHS Trust London United Kingdom; 3 Centre for Global Health and Equity, School of Health Sciences Swinburne University of Technology Hawthorn Australia; 4 Department of Public Health University of Copenhagen Copenhagen Denmark

**Keywords:** dermatology, health literacy, digital health literacy, digital literacy, skin, chronic, cluster analysis, innovation, eHealth literacy, dermatologists, telehealth, dermatologist, telemedicine, remote care, service, services, quality improvement

## Abstract

**Background:**

Given the expansion of remote digital dermatology services from the National Health Service, particularly during the COVID-19 pandemic, there is a need for methods that identify patients at risk of digital exclusion to guide equitable representation in service co-design processes and tailor remote services to the needs of their patient population.

**Objective:**

This quality improvement project aims to inform the redesign of remote services to optimally support the ongoing needs of patients with chronic skin diseases, ensuring that the services are tailored to patients’ digital health literacy requirements.

**Methods:**

We profiled the digital health literacy of 123 people with chronic skin conditions who require long-term surveillance in 2 specialist clinics (London, United Kingdom) using the Multidimensional Readiness and Enablement Index for Health Technology (READHY) questionnaire alongside the Optimizing Health Literacy and Access (Ophelia) process for hierarchical cluster analysis.

**Results:**

The cluster analysis of READHY dimensions in responding participants (n=116) revealed 7 groups with distinct digital and health literacy characteristics. High READHY scores in groups 1 (n=22, 19%) and 2 (n=20, 17.2%) represent those who are confident with managing their health and using technology, whereas the lower-scoring groups, 6 (n=4, 3.4%) and 7 (n=12, 10.3%), depended on traditional services. Groups 3 (n=27, 23.3%), 4 (n=23, 19.8%), and 5 (n=8, 6.9%) had varying digital skills, access, and engagement, highlighting a population that may benefit from a co-designed dermatology service.

**Conclusions:**

By identifying patient groups with distinguishable patterns of digital access and health literacy, our method demonstrates that 63.8% (n=74) of people attending specialist clinics in our center require support in order to optimize remote follow-up or need an alternative approach. Future efforts should streamline the READHY question profile to improve its practicality and use focus groups to elicit strategies for engaging patients with digital services.

## Introduction

Technological advances alongside the COVID-19 pandemic have driven remote digital dermatology service adoption across the National Health Service (NHS). Such services include application-based patient-initiated follow-up, where people initiate an appointment, as required, using their devices [1]. Our hospital department (London, United Kingdom) provides several specialist clinics for people under long-term dermatology follow-up. Considering many of these patients are not locals, a digital patient-initiated follow-up service may be an efficient and cost-effective alternative. However, this may widen inequities by disadvantaging digitally excluded individuals, including the estimated 10 million UK residents who have unequal access and capacity to use technologies that are essential for participating in society fully [2]. Specific patient-reported barriers associated with remote dermatology include low technology use, poor telephone facilities, and difficulty with photo sharing [3-5].

NHS England suggests several actions to mitigate digital exclusion, including creating guidance that measures teledermatology referral suitability [6]. Dermatology literature provides sparse information on the most appropriate measure. However, 1 way to determine this suitability is by measuring patients’ health technology readiness (how prepared and willing one is to use health technology) using the Multidimensional Readiness and Enablement Index for Health Technology (READHY) questionnaire [7]. In addition to exploring the mechanisms behind readiness, such as the motivation to engage with digital services, this tool identifies those at risk of digital exclusion [7]. Co-design with this population would provide the essential user-centered approach needed to develop a tailored service [8].

This study documents the initial steps of a quality improvement project, whereby we profile the health technology readiness of people for whom we plan to use digital services in the dermatology outpatient setting, aiming to use these data to optimize service design.

## Methods

### Recruitment

We invited consecutive people receiving long-term dermatology follow-up at 2 specialist clinics in our department—organ transplant recipient skin cancer surveillance (OTS) and biologics monitoring for chronic inflammatory skin disorders (BioM).

The READHY questionnaire is a validated tool based on the concept of digital health literacy, self-management, and social support using 13 related scales from the eHealth Literacy Questionnaire (7 scales) [9], Health Literacy Questionnaire (2 scales) [10], and Health Education Impact Questionnaire (4 scales) [11]. This tool assesses health technology readiness using 65 statements that participants respond to using a 4-point Likert scale (1=strongly disagree and 4=strongly agree). By averaging the scores for the responses to each question of a given scale, each scale is given an overall rating [7].

Authors (HM, AUP, and ZH) verbally administered the READHY questionnaire in the BioM (February-March 2022) and OTS (July-October 2021) clinics by telephone or in person, with assistance where required. Additional questions were asked to acquire demographic data, including age, gender, and ethnicity. People who could not understand basic spoken English were excluded.

### Analysis

In addition to descriptive statistics, the READHY responses and demographic data were subjected to cluster analysis using the Optimizing Health Literacy and Access (Ophelia) process [9]. Based on the principle of health equity, the Ophelia process recognizes that a population is not homogenous and there are subgroups within a population that may have different strengths and challenges, especially since health literacy or health technology readiness is a multidimensional concept. Hence, cluster analysis, a statistical method to identify subgroups based on a set of variables, is recommended. Following the Ophelia process protocol, a hierarchical cluster analysis using the Ward method, based on the 13 scale scores of the READHY tool, was undertaken. This helps to identify the strengths and challenges of subgroups among survey participants to foster the development of tailored actions to support the use of the service. People who did not answer at least 1 piece of demographic data were excluded from this analysis.

### Ethical Considerations

This work forms part of a quality improvement project and was approved by the local Quality Improvement Team (137292). Patients were invited to participate and provided informed verbal consent. The data was anonymized. No compensation was provided.

## Results

### Demographics

Of the 163 people (BioM: n=35, 21.5%; OTS: n=128, 78.5%) we invited to participate, 23 (66.5% response rate) out of 35 people from the BioM clinic and 100 (77.3% response rate) out of 128 people from the OTS clinic completed the questionnaire. There were 4 reasons for nonparticipation (40/163, 24.5%). Of the 163 people invited, 21 (12.9%) did not answer our telephone call, 14 (8.6%) declined our invitation, 3 (1.8%) did not telephone back, and 2 (1.2%) did not have sufficient English language skills. The final cohort (Table 1) consisted of 48 (BioM:OTS=11:37) women and 66 (BioM:OTS**=**12:54) men with a median age of 58.6 (IQR 50.2-66.6; BioM:OTS=52.6, IQR 38.9-60.1:60.1, IQR 51.7-67.6) years.

**Table 1:** Demographics of people included in this quality improvement project^a^.

Demographics		All (n=123), n (%)	Group 1 (n=22), n (%)	Group 2 (n=20), n (%)	Group 3 (n=27), n (%)	Group 4 (n=23), n (%)	Group 5 (n=8), n (%)	Group 6 (n=4), n (%)	Group 7 (n=12), n (%)
**Age group (years)**									
	21-29	2 (1.6)	0 (0)	0 (0)	0 (0)	0 (0)	0 (0)	1 (25)	1 (8.3)
	30-39	7 (5.7)	3 (13.6)	2 (10)	0 (0)	0 (0)	0 (0)	0 (0)	2 (16.7)
	40-49	17 (13.8)	4 (18.2)	5 (25)	5 (18.5)	2 (8.7)	0 (0)	0 (0)	1 (8.3)
	50-59	34 (27.6)	6 (27.3)	6 (30)	11 (40.7)	6 (26.1)	1 (12.5)	0 (0)	4 (33.3)
	60-69	35 (28.5)	6 (27.3)	3 (15)	6 (22.2)	11 (47.8)	6 (75)	1 (25)	2 (16.7)
	70-79	17 (13.8)	3 (13.6)	4 (20)	3 (11.1)	3 (13)	1 (12.5)	1 (25)	2 (16.7)
	80 or older	2 (1.6)	0 (0)	0 (0)	1 (3.7)	0 (0)	0 (0)	1 (25)	0 (0)
	Not answered	9 (7.3)	0 (0)	0 (0)	1 (3.7)	1 (4.3)	0 (0)	0 (0)	0 (0)
**Gender**									
	Men	67 (54.5)	15 (68.2)	11 (55)	15 (55.6)	12 (52.2)	6 (75)	1 (25)	7 (58.3)
	Women	48 (39)	7 (31.8)	9 (45)	12 (44.4)	11 (47.8)	1 (12.5)	3 (75)	5 (41.7)
	Prefer not to say	0 (0)	0 (0)	0 (0)	0 (0)	0 (0)	0 (0)	0 (0)	0 (0)
	Not answered	8 (6.5)	0 (0)	0 (0)	0 (0)	0 (0)	1 (12.5)	0 (0)	0 (0)
**Ethnicity**									
	Asian or British Asian	9 (7.3)	3 (13.6)	2 (10)	2 (7.4)	1 (4.3)	0 (0)	0 (0)	1 (8.3)
	Black, African, Caribbean, or Black British	9 (7.3)	0 (0)	3 (15)	1 (3.7)	1 (4.3)	1 (12.5)	0 (0)	3 (25)
	White	95 (77.2)	19 (86.4)	14 (70)	23 (85.2)	21 (91.3)	7 (87.5)	4 (100)	7 (58.3)
	Mixed or multiple ethnicity groups	2 (1.6)	0 (0)	0 (0)	1 (3.7)	0 (0)	0 (0)	0 (0)	1 (8.3)
	Prefer not to say	1 (0.8)	0 (0)	1 (5)	0 (0)	0 (0)	0 (0)	0 (0)	0 (0)
	Not answered	7 (5.7)	0 (0)	0 (0)	0 (0)	0 (0)	0 (0)	0 (0)	0 (0)
**Marital status**									
	Married	73 (59.3)	13 (59.1)	14 (70)	20 (74.1)	17 (73.9)	6 (75)	1 (25)	2 (16.7)
	Divorced	7 (5.7)	1 (4.5)	1 (5)	2 (7.4)	0 (0)	0 (0)	1 (25)	2 (16.7)
	Widowed	5 (4.1)	0 (0)	1 (5)	1 (3.7)	2 (8.7)	0 (0)	1 (25)	0 (0)
	Never married	15 (12.2)	5 (22.7)	0 (0)	2 (7.4)	1 (4.3)	1 (12.5)	1 (25)	5 (41.7)
	Separated	5 (4.1)	1 (4.5)	3 (15)	0 (0)	0 (0)	0 (0)	0 (0)	1 (8.3)
	Engaged	2 (1.6)	1 (4.5)	0 (0)	0 (0)	1 (4.3)	0 (0)	0 (0)	0 (0)
	Partner	2 (1.6)	0 (0)	0 (0)	0 (0)	0 (0)	1 (12.5)	0 (0)	1 (8.3)
	Not answered	14 (11.4)	1 (4.5)	1 (5)	2 (7.4)	2 (8.7)	0 (0)	0 (0)	1 (8.3)
**Education**									
	Primary school	1 (0.8)	0 (0)	0 (0)	0 (0)	1 (4.3)	0 (0)	0 (0)	0 (0)
	Secondary school (up to 16 years)	36 (29.3)	6 (27.3)	7 (35)	5 (18.5)	12 (52.2)	2 (25)	1 (25)	3 (25)
	College or university (ie, bachelor’s degree)	35 (28.5)	11 (50)	2 (10)	11 (40.7)	1 (4.3)	5 (62.5)	2 (50)	3 (25)
	Higher or further education (ie, A-levels)	24 (19.5)	2 (9.1)	5 (25)	7 (25.9)	6 (26.1)	1 (12.5)	0 (0)	3 (25)
	Postgraduate degree	10 (8.1)	2 (9.1)	4 (20)	1 (3.7)	1 (4.3)	0 (0)	1 (25)	1 (8.3)
	Prefer not to say	5 (4.1)	0 (0)	1 (5)	1 (3.7)	2 (8.7)	0 (0)	0 (0)	1 (8.3)
	Not answered	12 (9.8)	1 (4.5)	1 (5)	2 (7.4)	0 (0)	0 (0)	0 (0)	1 (8.3)
**Household income (£)^b^**									
	Up to 17,499	24 (19.5)	3 (13.6)	7 (35)	2 (7.4)	5 (21.7)	0 (0)	2 (50)	5 (41.7)
	17,500-29,999	13 (10.6)	3 (13.6)	1 (5)	2 (7.4)	3 (13)	1 (12.5)	1 (25)	2 (16.7)
	30,000-39,999	6 (4.9)	0 (0)	1 (5)	1 (3.7)	2 (8.7)	2 (25)	0 (0)	0 (0)
	40,000-49,999	9 (7.3)	2 (9.1)	0 (0)	4 (14.8)	2 (8.7)	0 (0)	0 (0)	1 (8.3)
	50,000 and over	32 (26)	10 (45.5)	5 (25)	9 (33.3)	2 (8.7)	4 (50)	0 (0)	2 (16.7)
	Not answered	39 (31.7)	4 (18.2)	6 (30)	9 (33.3)	9 (39.1)	1 (12.5)	1 (25)	2 (16.7)

^a^People who did not answer 1 or more demographic questions were excluded from the cluster analysis.

^b^1 £=US $1.22.

### Outcomes

**Figure 1:**
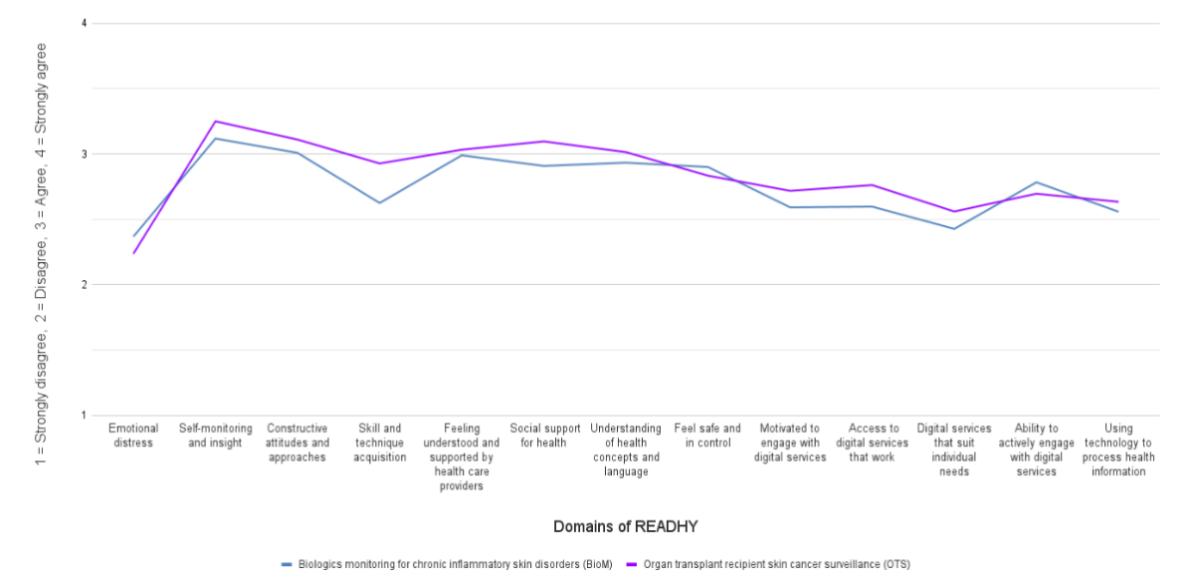
Mean READHY domain scores as per clinic. READHY: Multidimensional Readiness and Enablement Index for Health Technology.

The mean READHY domain scores followed a similar trend in both clinics (Figure 1), with higher scores for self-monitoring, support, and health understanding and lower scores for emotional distress, suitability, and technology for processing health information. The most notable difference in domain responses occurred in “skills and technique acquisition,” where the OTS group scored higher.

**Figure 2:**
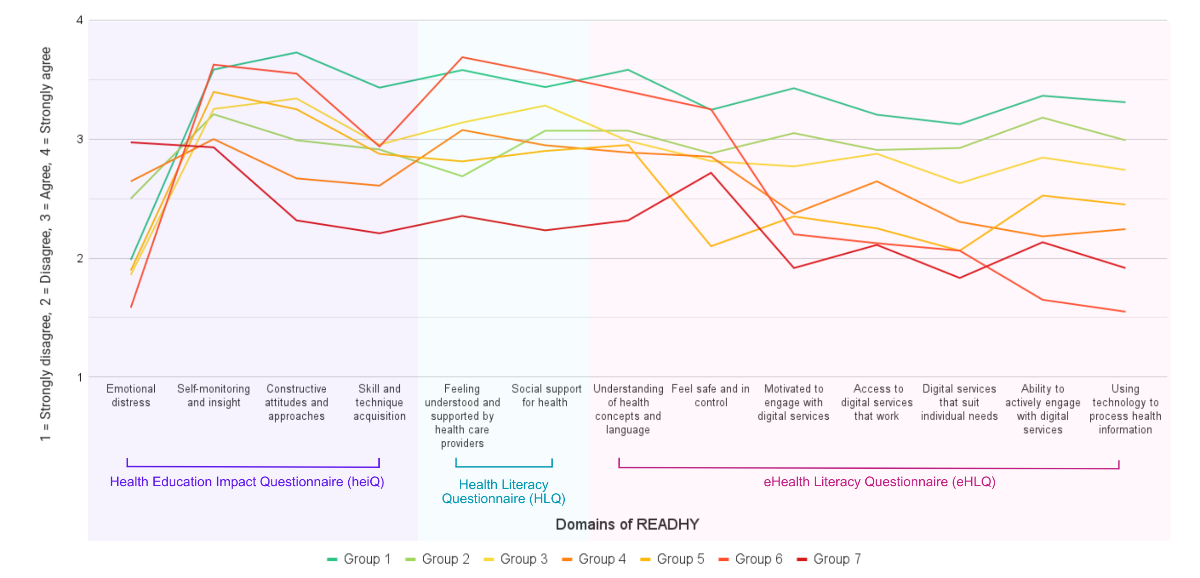
Mean READHY domain scores as per cluster analysis. READHY: Multidimensional Readiness and Enablement Index for Health Technology.

**Figure 3:**
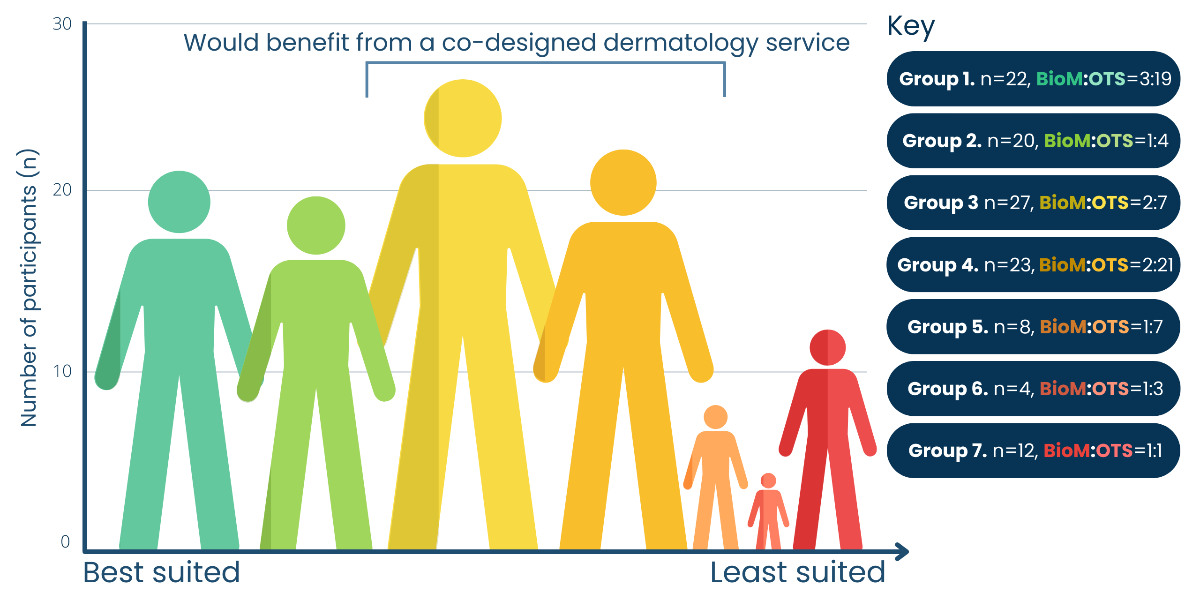
Patient groups (1-7) based on hierarchical cluster analysis of demographics and READHY scores. BioM: biologics monitoring for chronic inflammatory skin disorders; OTS: organ transplant recipient skin cancer surveillance; READHY: Multidimensional Readiness and Enablement Index for Health Technology.

A total of 116 people were eligible for the cluster analysis, which revealed 7 groups (Table 1 and Figures 2 and 3). The higher READHY scores in groups 1 (n=22, 19%; median age 56.2, IQR 45.8-65.3 y; men:women=15:7) and 2 (n=20, 17.2%; median age 54.5, IQR 45.5-66.2 y; men:women=11:9) represented those confident with managing their health and using technology, although people in group 2 reported somewhat higher emotional distress. Conversely, groups 6 (n=4, 3.4%; median age 69.5, IQR 29.5-79.5 y; men:women=1:3) and 7 (n=12, 10.3%; median age 54.5, IQR 39.5-64.5 y; men:women=7:5) were low-scoring populations dependent on traditional services with limited access to and engagement with prospective remote care. Accompanying their low digital health literacy, group 7 members felt less supported, more emotionally distressed, and with a lower sense of control. Groups 3 (n=27, 23.3%; median age 56.8, IQR 50.9-65.3 y; men:women=5:4), 4 (n=23, 19.8; median age 62.2, IQR 55.3-67.2 y; men:women=9:5), and 5 (n=8, 6.9%; median age 64.5, IQR 61.2-67.8 y; men:women=6:1) consisted of well-supported individuals possessing some experience with digital services. However, each group had varying levels of access to, interest in, and skills in using technology for health management.

## Discussion

### Principal Findings

We use a method in a cohort of people receiving long-term dermatology follow-up revealing that 63.8% (74/116) of these individuals belong to groups 3 to 7, which are characterized by lower health technology readiness and are vulnerable to digital exclusion. However, members of groups 3 to 5 have moderate health technology experience, alongside support in the community, highlighting a population that may use a co-designed dermatology service. Additionally, lower readiness was not associated with any specific demographics. It is, therefore, essential to evaluate health technology readiness when developing remote dermatology services to recognize those that may already safely benefit from technology (groups 1 and 2), require targeted support (groups 3 to 5), or need alternative care provision (groups 6 and 7).

Stratifying health technology readiness has only ever been successfully conducted outside of a dermatological setting, such as in an outpatient irritable bowel disease clinic [12]. Furthermore, there is minimal literature exploring user suitability for digital dermatology care. eHealth literacy has, however, previously been assessed by Stege et al [13] in a population of patients with skin cancer. Stege et al [13] report greater eHealth literacy in younger, well-educated participants, though we are unable to determine from their data the proportion of their participants who are at risk of digital exclusion. Our comparative lack of demographic trends may be due to the broad inclusion criteria for the cluster analysis and limited sample size.

Unexpectedly, most of our cohort fell within groups 1 to 4, with group 1 being the third largest cluster overall. This skew of our population toward profiles with higher health technology readiness could be explained by the upskilling of the public during the COVID-19 pandemic, thus improving their confidence with digital tools. Nielsen et al [12] and Thorsen et al [14] document a similar skew, although this is minimal in Thorsen et al [14] perhaps due to the data collection that occurred in 2018, before the previously mentioned upskilling.

### Limitations

The limitations include using direct data collection alongside self-reported demographics. Social desirability bias may affect our findings since participants may not want to reveal information that is more sensitive. Indeed, the 31.7% (39/123) of eligible participants who did not disclose their household income supports this notion. Next, despite an acceptable response rate, sample selection bias is likely present since we used a highly comprehensive measure of health technology readiness and a translator was absent, excluding non-English speakers and those with low literacy. Finally, the generalization of our findings to the wider population requiring long-term dermatology follow-up is limited as we surveyed only 2 clinics. Clinical interviews and focus groups with people who are frequently difficult to engage in will need to be a part of future work to elicit their opinions about digital health interventions. Furthermore, streamlining the READHY question profile would likely improve its practicality in busy clinical settings.

### Conclusions

In summary, through a preliminary exploration of READHY, we demonstrate that 63.8% (74/116) of people attending specialist clinics in our center need at least some support to optimize remote digital follow-up. This proportion is likely to vary considerably across centers and patient populations. However, it is paramount that clinicians consider such information to guide equitable representation in service co-design processes and tailor remote services to the needs of their patient population.

## Abbreviations

BioM: biologics monitoring for chronic inflammatory skin disorders
NHS: National Health Service
Ophelia: Optimizing Health Literacy and Access
OTS: organ transplant recipient skin cancer surveillance
READHY: Multidimensional Readiness and Enablement Index for Health Technology
